# Development and validation of a novel prediction model for hypertensive disorders of pregnancy based on maternal cardiovascular function and placental blood flow metrics at 22 to 24 gestational weeks using machine learning

**DOI:** 10.1038/s41598-025-30738-3

**Published:** 2025-12-09

**Authors:** Juulia Lantto, Kari Flo, Åse Vårtun, Christian Widnes, Jonas Johnson, Ganesh Acharya

**Affiliations:** 1 Division of Obstetrics & Gynecology, Department of Clinical Science, Intervention and Technology (CLINTEC), Karolinska Institutet, Stockholm, Sweden; 2 Department of Clinical Medicine, UiT-The Arctic University of Norway, Tromsø, Norway; 3 Division of Obstetrics and Gynecology, Akershus University Hospital, Lørenskog, Norway; 4 Department of Gynecology and Obstetrics, Vestre Viken Hospital Trust, Baerum Hospital, Baerum, Norway

**Keywords:** Hypertensive disorders of pregnancy, Maternal hemodynamics, Prediction model, Umbilical artery, Uterine artery, Machine learning, Diseases, Health care, Risk factors

## Abstract

Hypertensive disorders of pregnancy (HDP) remain a leading cause of maternal and perinatal morbidity worldwide, and current screening strategies have limited predictive value in low-risk populations especially for late-onset HDP. This study aimed to develop and internally validate an interpretable machine-learning model for predicting HDP using noninvasive parameters of maternal systemic hemodynamics, endothelial function, and utero-placental and feto-placental blood flow measured in mid-pregnancy. In this cross-sectional cohort, 577 normotensive women underwent impedance cardiography and Doppler ultrasonography at 22^+ 0^ to 23^+ 6^ weeks’ gestation. The incidence of HDP was 16.6% (96/577) most (87.5%) occurring at term (≥ 37 weeks), including 73 cases of gestational hypertension (12.6%) and 23 cases of pre-eclampsia (4.0%). An optimized predictive model with seven physiological features was developed using Extreme Gradient Boosting (XGBoost). Hyperparameters were optimized using stratified 10-fold cross-validation maximizing average Precision Recall Area Under the Curve (PR–AUC), and the decision threshold was selected by maximizing the geometric mean of sensitivity and specificity on a separate validation set. On an independent test set (*n* = 58; 10 HDP), the model achieved an Area Under the Receiver Operating Curve (ROC–AUC) of 0.82 (95% CI 0.65–0.95) and a PR–AUC of 0.57 (95% CI 0.26–0.84). At the optimized operating point, sensitivity was 70% (95% CI 0.40–1.00), specificity 79% (95% CI 0.67–0.90), precision 41% (95% CI 0.18–0.65), and negative predictive value 93% (95% CI 0.84–1.00). This interpretable, non-invasive mid-gestation model demonstrates strong discrimination and excellent negative predictive value, supporting its integration into routine second-trimester screening for risk stratification without reliance on biochemical markers.

**Supplementary Information:**

The online version contains supplementary material available at 10.1038/s41598-025-30738-3.

## Introduction

The hypertensive disorders of pregnancy (HDP) are a common pregnancy complication associated with adverse maternal and perinatal outcomes^1,2^. The reported prevalence of HDP is approximately 4-5.9% of livebirths in the Nordic countries^3–6^ but much higher (∼10%) globally. The pathophysiology of HDP is acknowledged to be heterogenous with maternal, placental and fetal contributions^7–11^.

The clinical risk factors for developing HDP have been identified by several large-scale studies^12–18^. The major risk factors include HDP during previous pregnancy, chronic kidney disease, chronic hypertension, autoimmune disease (e.g. antiphospholipid syndrome, systemic lupus erythromatosus), pregestational diabetes mellitus and the moderate risk factor are nulliparity, age ≥ 40 years, interpregnancy interval > 0 years, body-mass index BMI $\:35\:$kg/m^2^, family history of pre-eclampsia (PE), and multifetal pregnancy^17^.

Combinations of several clinical characteristics with ultrasonographic and biochemical markers during first and second trimesters of pregnancy have been studied to predict which women develop gestational hypertension and/or PE later in pregnancy^7^. Many of these have not been sufficiently effective for screening asymptomatic, low-risk women due to high negative and low positive predictive values^19,20^. The recent focus has been on first trimester screening due to the possibility of starting prophylactic treatment with Aspirin before 15–16 weeks of gestation, which is shown to be effective in preventing early onset disease^21^. However, the prevalence and relative burden of late-onset/term HDP is substantially higher than that of the early-onset/preterm PE with regards to maternal and perinatal complications including mortality^22^. Unfortunately, even the multivariate tools have a low (approximately 40% − 50%) detection rate for predicting late-onset HDP^7,13,15^. Therefore, an improved prediction method could help to reduce morbidity through better stratification of risk, surveillance and appropriate timing of delivery avoiding serious complications.

Impaired maternal cardiovascular hemodynamics, endothelial and placental function have been implicated as the factors associated with the development of HDP, and several studies have evaluated their role in screening HDP individually. However, to the best of our knowledge, no screening study has used a combined assessment of maternal cardiac function, systemic hemodynamics, endothelial function and utero-placental and feto-placental blood flow at a single point in gestation in the same study population for predicting HDP.

At gestational week 22–24, the placentation is fully established^23,24^, fetal abnormalities are excluded by routine morphological ultrasound scanning, maternal cardiovascular adaptation to pregnancy is mostly complete and maternal blood pressure is at its lowest^25^. Thus, we hypothesized that a combined assessment of maternal cardiovascular function and placental blood flow after mid-gestation, together with maternal characteristics and medical history, could predict term HDP better compared to previous studies conducted earlier in gestation before full maturation of the placenta, or those including maternal biochemical marker analyses. We employed a machine learning approach to optimize the predictive model.

## Methods

This cross-sectional study was conducted at the University Hospital of North Norway, Tromsø, from 2006 to 2015. Pregnant women attending the antenatal clinic for routine second trimester ultrasonography at 17^+ 0^-19^+ 6^ gestational weeks were informed about the study and invited to participate. Inclusion criteria were normotensive pregnant women, age > 18 years, gestational age confirmed by ultrasound before 20 weeks, and uncomplicated singleton pregnancy without any fetal or placental malformations. Exclusion criteria were; multifetal pregnancy, presence of any major fetal congenital structural or chromosomal abnormality and pre-existing medical conditions with a known high risk for HDP, such as hypertension, chronic renal disease, pregestational diabetes mellitus, and antiphospholipid antibody syndrome. All women agreeing to participate in the study signed a written informed consent. The study was approved by the Regional Committee for Medical and Health Research Ethics (REK-Nord, reference number 5.2005.1386). All clinical procedures and research methods were performed in accordance with the relevant guidelines and regulations.

### Maternal anthropometry

Participants were examined at 22 + 0 to 23 + 6 gestational age. Height was measured using an altimeter (Charder Electronic Co, Taichung City, Taiwan) and bodyweight was measured using an electronic scale (Soehnle, Leifheit AG, Nassau, Germany). Body mass index (BMI) was calculated as weight/height^2^ and the body surface area (BSA) was calculated as = 0.007184 x Height ^0.725^ x Weight ^0.425^^26^.

### Assessment of maternal cardiac function and systemic hemodynamics

Maternal cardiac function and systemic hemodynamics were assessed using impedance cardiography (ICG) (Philips Medical Systems, Androver, MA, USA) after 10–15 min of rest in a supine semi-recumbent position as previously described in detail^27^.

The following variables were directly measured and displayed on the ICG screen: heart rate (HR), stroke volume (SV), systolic blood pressure (SBP), diastolic blood pressure (DBP), pre-ejection period (PEP), left-ventricular ejection time (LVET), thoracic fluid content (TFC), velocity index (VI) and accelerated cardiac index (ACI). The following variables were calculated as follows: cardiac output (CO) = SV x HR, cardiac index (CI) = CO/BSA, mean arterial pressure (MAP) = DBP + 1/3 (SBP-DBP), systemic vascular resistance (SVR) = ((MAP-CVP)/CO) x 80, systemic vascular resistance index (SVRI) = SVR/BSA, systolic time ratio (STR) = PEP/LVET, left cardiac work index (LCWI) = (MAP-PAOP) x CO/BSA x 0.0144.

### Assessment of maternal endothelial function

Maternal endothelial function was assessed with NO-dependent flow-mediated vasodilatation (FMD). The right brachial artery diameter was measured using two-dimensional B-mode ultrasound approximately 5 cm above the antecubital fossa using an 8-MHz linear transducer (Acuson Sequoia 512, Mountain View, CA, USA) following the International Brachial Artery Reactivity Task Force guidelines^28^ as previously described in detail^29^. Following the baseline measurements, an inflatable pressure cuff was placed on the upper arm approximately 8 cm above the elbow and inflated until the pressure was 50 mmHg above the systolic blood pressure and maintained for 5 min. The inner diameter of the brachial artery was measured in a magnified B-mode grey scale ultrasound image in the end-systole (incident with the peak T-wave of the ECG) 60 s after the cuff release and the change in brachial artery diameter from the baseline value (ΔBADIA) was calculated.

### Measurement utero-placental blood flow

The uterine artery (UtA) blood flow velocities were measured transabdominally using Doppler ultrasonography with a 6-MHz curvilinear transducer (Acuson Sequoia 512, Mountain View, CA, USA) as previously described^30^. Color-directed pulsed-wave Doppler was used to measure the time-averaged velocity (UtA_TAV_), peak systolic velocity (UtA_Max_), end-diastolic velocity (UtA_Min_), and time-averaged maximum velocity (UtA_TAMXV_) from the left and right UtA. An average of three consecutive waveforms was used for analysis. Power Doppler angiography was used to measure the uterine artery diameter. The uterine artery volume blood flow (Q_UtA_) was calculated as: UtA time-averaged intensity weighted mean velocity × cross-sectional area of the UtA. The cross-sectional area of UtA was calculated as: 3.14 × (UtA diameter/2)^2^.

The uterine artery pulsatility index (UtA PI) was calculated as: (peak systolic velocity – end diastolic velocity)/ time-averaged maximum velocity, the uterine artery resistance index (UtA RI) as: (peak systolic velocity – end diastolic velocity)/peak systolic velocity, and the uterine artery vascular resistance (*R*_uta_) (mmHg/mL/min) was calculated as: MAP/*Q*_UtA_. The fraction of maternal CO supplying the utero-placental circulation was calculated as: *Q*_UtA_ /CO x 100. Mean values of both uterine arteries were used for statistical analysis.

### Measurement of feto-placental blood flow

Feto-placental blood flow was assessed using Doppler ultrasonography of umbilical artery (UA) and umbilical vein (UV) as described previously^31,32^. The UA blood flow velocity waveforms were obtained from a free-floating loop of the umbilical cord using color-directed pulsed-wave Doppler to measure the time-averaged velocity (UA_TAV_), peak systolic velocity (UA_Max_), end-diastolic velocity (UA_Min_), and time-averaged maximum velocity (UA_TAMXV_) from the umbilical artery, and an average value of three consecutive waveforms in the absence of fetal or maternal movements was recorded.

The UA PI was calculated as (peak systolic velocity – end-diastolic velocity)/time-averaged maximum velocity. The resistance index (UA RI) was calculated as (peak systolic velocity – end-diastolic velocity)/ peak systolic velocity. UA acceleration time (UA AT) was calculated as the time difference between the onset to peak of UA_Max_. All these measurements were automatically calculated using the software of the ultrasound machine and displayed on screen.

The UV time-averaged maximum velocity and the inner diameter of the vessel were measured at a free loop of umbilical cord^33^. Blood flow velocity was measured during fetal quiescence for 2–4 s using a large (5–10 mm) sample volume.

The umbilical venous volume blood flow (Q_uv_) was calculated as: 0.5 × time-averaged maximum velocity × π × (UV diameter/2)^2^, assuming a parabolic velocity profile and circular cross-section of the vessel, and normalized for the estimated fetal weight (EFW) calculated using Hadlock formula (Log_10_ weight = 1.335–0.0034 AC x FL + 0.0316 BPD + 0.0457 AC + 0.1623 FL)^34^. Normalized Q_UV_ was calculated as: norm-Q_UV_ = Q_UV_/EFW).

For all blood flow velocity measurements with ultrasound, the angle of insonation was kept close to zero and always < 15° for Doppler recordings, and close to 90° for the measurements of vessel diameter. To ensure the safety of the ultrasonography, the ALARA (as low as reasonably achievable) principle was used, total scanning time never exceeded 60 min, and the guidelines of the International Perinatal Doppler Society^35^ were followed during Doppler sonographic examinations. The mechanical index was kept below 1.9 and the thermal index below 1.5, and in most of the sessions, they were below 1.0.

### Follow up of participants and outcomes

Maternal characteristics were obtained from electronic medical records. Maternal weight, height, systolic and diastolic blood pressure values were recorded at the first visit to the maternal health clinic at early gestation. The participating women were followed prospectively, and course and outcome of pregnancy were recorded.

Gestational hypertension was defined as new-onset non‐proteinuric hypertension (blood pressure ≥ 140/90 mmHg), and PE as new‐onset hypertension with proteinuria (≥ 1 + on a routine urinalysis or ≥ 300 mg/24 hour) or signs of organ or placental dysfunction (i.e. maternal acute kidney injury, liver dysfunction, neurological symptoms, hemolysis and thrombocytopenia, or fetal growth restriction) after 20 weeks of gestation. HDP was defined as the presence of either gestational hypertension or PE.

Gestational age at birth, mode of delivery, sex of the neonate, birth weight, placental weight and Apgar score were obtained from the electronic medical records. Small for gestational age (SGA) was defined as birthweight < 10 percentile using Nordic reference standards^36^. All neonates were examined at least once by a pediatrician before being discharged from the hospital.

### Data processing and model building

The initial dataset consisted of 638 samples, including 116 women who developed HDP. Rows containing more than 7.5% missing values were excluded, yielding a final dataset of 577 participants comprising 96 HDP (minority) and 481 normotensive (majority) cases. Outliers were identified using two complementary approaches: the Interquartile Range (IQR) method (multiplier > 1.5) and z-score filtering (threshold > 3.0). Following outlier removal, missing values both original and those introduced through outlier handling, were imputed using K-Nearest Neighbors (KNN), *k* = 5. To address the pronounced class imbalance (96 HDP vs. 481 normotensive), the dataset was partitioned into training (80%), validation (10%), and test (10%) using stratified sampling. The Adaptive Synthetic Sampling (ADASYN) algorithm was applied exclusively to the training set, which originally contained 462 samples (76 minority, 386 majority). After resampling, the training set was balanced to 778 samples (385 original majority and 393 synthetically generated minority instances). The validation (58 samples: 10 minority, 48 majority) and test sets (58 samples: 10 minority, 48 majority) retained their original class distributions, enabling unbiased model evaluation.

For subsequent analyses, features were grouped into clinically interpretable domains: maternal baseline characteristics and obstetric history; maternal endothelial function assessed by FMD; utero-placental and feto-placental blood-flow parameters measured by Doppler ultrasonography; and maternal cardiac function and systemic hemodynamics assessed using ICG.

Feature selection was performed in a two-step process. Initially, 43 candidate predictors were included (see Supplementary Table S1 for full variable definitions). Model performance was evaluated iteratively using receiver operating characteristic area under the curve (ROC–AUC), false negative rates, and clinical interpretability. Based on both statistical contribution and physiological plausibility, seven features were retained as the final predictor set: maternal SBP, CO, SVR, UA AT, norm-QUV, total Q_UtA_, and mean UtA PI. This approach ensured that the final model combined strong discriminative ability with clear physiological interpretability.

All analyses were conducted in Python 3.13 using scikit-learn 1.6.1, XGBoost 3.0.5, CatBoost 1.2.8, LightGBM 4.6.0, Matplotlib 3.10.3, and SHAP 0.48.0. A structured scikit-learn pipeline was implemented encompassing data preprocessing, feature selection, hyperparameter optimization, and independent test evaluation. Extreme Gradient Boosting (XGBoost) was selected as the primary classifier owing to its robustness and established performance in structured biomedical datasets. For benchmarking, CatBoost, LightGBM, and logistic regression models were trained under identical preprocessing and tuning procedures (full details in Supplementary Table S3). Hyperparameter optimization was performed using RandomizedSearchCV with stratified 10-fold cross-validation (200 randomized configurations per model), optimizing the mean precision–recall area under the curve (PR–AUC) on the training folds. XGBoost achieved the most stable and discriminative cross-validated performance and was therefore retained as the final model. To reflect screening priorities under class imbalance and facilitate clinical interpretability, the operating threshold was selected on the validation set by maximizing the geometric mean (G-mean) of sensitivity and specificity. This threshold was then applied unchanged to the independent test set.

Final model performance was evaluated using accuracy, precision, recall, specificity, F1-score, Matthews correlation coefficient (MCC), Brier score, ROC–AUC, and PR–AUC. Statistical uncertainty was estimated via nonparametric bootstrap resampling (1,000 iterations) to derive 95% confidence intervals, providing an internal measure of model stability given the limited number of outcome events (*n* = 96 overall; *n* = 10 in the test set). For reproducibility, random seeds were fixed, software versions were pinned, and test data were strictly excluded from all stages of training, hyperparameter tuning, and threshold optimization. To enhance clinical interpretability, Shapley Additive Explanations (SHAP) were used to quantify each predictor’s contribution to the model’s output. SHAP dependence plots were generated for the most influential features, illustrating how variations in individual physiological parameters affect the predicted risk of HDP.

## Results

### Study population

Of a total of 638 women that were recruited and examined, 577 women were included for the final analysis. 61 were excluded due to missing data either due to incomplete measurements or missing final pregnancy outcomes.

### Baseline characteristics

The baseline characteristics of the study population are presented in Table 1. At the first antenatal booking visit, 1.4% (8/577) of the women were over 40 years of age, and 0.7% (4/577) of the women had BMI ≥ 35 kg/m^2^. There were significant differences in mean BMI and blood pressures (SBP, DBP and MAP) in early pregnancy (first trimester booking visit) between women who developed HDP compared to those who remained normotensive (Table 1).

**Table 1:** Maternal characteristics at booking.

Parameter	All women, *N* = 577				Women with HDP, *N* = 96				Women without HDP, *N* = 461				Difference between groups
	Mean	SD	95% CI, lower limit	95% CI, upper limit	Mean	SD	95% CI, lower limit	95% CI, upper limit	Mean	SD	95% CI, lower limit	95% CI, upper limit	*p*-value
Age (years)	29.4	4.8	29.0	30.0	30.1	4.8	29.1	31.1	29.2	4.8	28.8	29.7	0.108
BMI (Kg/m^2^)	25.68	3.42	25.40	25.96	26.61	3.55	25.89	27.33	25.49	3.37	25.19	25.79	0.006
Systolic blood pressure (mmHg)	116	10	115	117	121	11	119	123	115	10	114	116	< 0.001
Diastolic blood pressure (mmHg)	70	8	69	70	73	9	71	75	69	8	68	70	< 0.001
New partner in current pregnancy	9.2% (53)				9.4% (9)				9.5% (44)				0.944
Smoking	5.2% (30)				4.2% (4)				5.6% (26)				0.616
Use of ART	2.4% (14)				1.0% (1)				2.8% (13)				0.297

Data are presented as mean, standard deviation, 95% confidence intervals (CI) and difference between groups (women developing HDP vs. not developing HDP).

Abbreviations: ART, assisted reproductive technology; BMI, body mass index; CI, confidence interval; HDP, hypertensive disorders of pregnancy; SD, standard deviation.

### Obstetric history

Obstetric history of the study participants is presented in Table 2. Of all women, 48.4% (279/577) were nullipara. 46.9% (45/96) of women developing HDP and 50.8% (234/461) of women who did not develop HDP were nullipara (no difference between groups, *p* = 0.751). There were no significant differences between those women who developed HDP compared to those who did not, except for the personal and family history of PE. Prevalence of history of PE in own previous pregnancy or family history of PE was 15.1% (87/577) in all women, 30.2% (29/96) in women developing HDP and 12.6% (58/461) in women without HDP.

**Table 2:** Obstetric history of the study population.

	All women *N* = 577	Women with HDP *N* = 96	Women without HDP *N* = 461	Difference between groups *P*-value
Nullipara	48.4% (279)	46.9% (45)	50.8% (234)	0.751
Previous livebirth	47.7% (275)	45.8% (44)	50.1% (231)	0.895
Previous miscarriage or abortion	33.1% (191)	35.4% (34)	34.1% (157)	0.601
Previous stillbirth	1.0% (6)	2.1% (2)	0.9% (4)	0.270
Pre-eclampsia in previous pregnancy	6.9% (40)	15.6% (15)	5.4% (25)	< 0.001
Family history of pre-eclampsia	9.7% (56)	16.7% (16)	8.7% (40)	0.012

Data are presented as mean, standard deviation, 95% confidence intervals and difference between groups (women developing HDP vs. not developing HDP).

Abbreviations: HDP, hypertensive disorders of pregnancy.

### Maternal medical history

Ten women reported to have allergy/asthma, six had had endocrine disorders (four hypothyroidism, one Greve´s disease and one Addison`s disease), three psoriasis, one hemochromatosis, one fibromyalgia, one epilepsy and one idiopathic thrombocytopenia. Six women had a history of inflammatory bowel disease, two had psychiatric disorders, four had migraines, two had polycystic ovarian syndrome, and two had endometriosis.

### Maternal cardiovascular function and placental function

Data on maternal endothelial function at 22 + 0–23 + 6 weeks of gestation measured by FMD, cardiac function and systemic hemodynamics measured by ICG, and utero-placental and feto-placental blood flow measured by Doppler ultrasonography are summarized in Table 3 and in entirety in Supplementary Table S1. There were significant differences in systolic (SBP), diastolic (DBP) and mean (MAP) blood pressures, BMI, umbilical artery acceleration time (UAAT), umbilical vein diameter (UV_Diam_), umbilical venous blood flow normalized for estimated fetal weight (norm-Q_UV_ (ml/min)), maternal CO, cardiac index (CI), stroke volume (SV) and left ventricular ejection time (LVET) parameters between the groups (Table 3, S1).

**Table 3:** Summary of the original data on maternal endothelial function, cardiac function and systemic hemodynamics, utero-placental and feto-placental hemodynamics measured at 22 + 0–23 + 6 weeks of gestation. (Full dataset available in the supplementary table S1).

Parameter	All women, *N* = 577				Women with HDP, *N* = 96				Women without HDP, *N* = 461				Difference between groups
	mean	SD	95% CI, lower limit	95% CI, upper limit	mean	SD	95% CI, lower limit	95% CI, upper limit	mean	SD	95% CI, lower limit	95% CI, upper limit	*p*-value
Maternal baseline characteristics at 22 + 0–23 + 6 weeks													
Systolic BP (mmHg)	101	9	101	102	108	9	106	110	100	8	99	101	< 0.001
Diastolic BP (mmHg)	68	7	67	68	72	7	71	74	70	7	66	67	< 0.001
Mean arterial pressure (mmHg)	79	7	78	80	84	7	83	86	78	7	77	79	< 0.001
BMI (Kg/m^2^)	23.94	3.34	23.66	24.21	24.81	3.67	24.06	25.55	23.76	3.25	23.47	24.05	0.007
Maternal endothelial function at 22 + 0–23 + 6 weeks													
ΔBADIA (mm)	0.04	0.03	0.03	0.04	0.04	0.02	0.03	0.04	0.04	0.03	0.03	0.04	0.583
Utero-placental blood flow measurements at 22 + 0–23 + 6 weeks													
UtA PI	0.78	0.19	0.76	0.80	0.81	0.21	0.77	0.86	0.77	0.19	0.76	0.79	0.088
UtA RI	0.51	0.08	0.50	0.51	0.52	0.09	0.50	0.54	0.50	0.08	0.50	0.51	0.108
UtAAT (s)	0.11	0.02	0.11	0.11	0.11	0.02	0.11	0.11	0.11	0.02	0.11	0.12	0.107
Q_UtA_ (ml/min)	522.5	287.0	499.1	546.0	560.3	303.4	498.8	621.7	515.0	283.4	489.6	540.4	0.299
Feto-placental blood flow measurements at 22 + 0–23 + 6 weeks													
UA PI	1.17	0.14	1.16	1.18	1.16	0.14	1.13	1.19	1.18	0.14	1.16	1.19	0.205
UA RI	0.72	0.05	0.71	0.72	0.71	0.05	0.71	0.72	0.72	0.05	0.72	0.72	0.349
UAAT (s)	0.09	0.02	0.09	0.10	0.09	0.02	0.09	0.09	0.10	0.02	0.09	0.10	0.001
norm-Q_UV_ (ml/min per kg EFW)	138.2	42.3	134.7	141.6	148.3	39.1	140.4	156.2	136.2	42.6	132.4	140.0	0.004
Maternal cardiac function and systemic hemodynamics at 22 + 0–23 + 6 weeks													
CO (L/min)	6.02	1.20	5.92	6.12	6.42	1.19	6.18	6.66	5.94	1.19	5.83	6.05	< 0.001
CI (L/min/m^2^)	3.33	0.51	3.28	3.37	3.48	0.51	3.38	3.59	3.30	0.50	3.25	3.34	0.003
SVR (dyne s/cm^5^)	1027.4	194.6	1011.4	1043.3	1023.13	193.67	983.88	1062.37	1028.19	194.98	1010.72	1045.66	0.570

Data are presented as mean, standard deviation, 95% confidence intervals and difference between groups (women developing HDP vs. not developing HDP). Values presented for all women, women developing HDP and not developing HDP, respectively.

Abbreviations: BMI, body mass index; BP, blood pressure; CI, cardiac index; CO, cardiac output; HDP, hypertensive disorders of pregnancy; Q_UtA_, total uterine artery blood flow; norm-Q_UV_, volume blood flow in umbilical vein normalized for estimated fetal weight; SVR, systemic vascular resistance; UA PI, umbilical artery pulsatility index; UA RI, umbilical artery resistance index; UAAT, umbilical artery acceleration time; UtA PI, uterine artery pulsatility index; UtA RI, uterine artery resistance index; ΔBADIA, change in brachial artery diameter during FMD.

### Pregnancy outcomes

The mean (SD) gestational age at birth was 281 (13) days (range 178–300), mean birth weight was 3533 (548) g /range 700–5222 g, placental weight 619 (136) g, umbilical artery pH 7.23 (0.09), umbilical artery base excess (BE) -4.7 (3.4), umbilical vein pH 7.25 (0.09), umbilical vein BE -4.9 (3.0), and Apgar score below < 7 at 5 min in 2.4%. 54.2% of the newborns were male and 45.8% female. The onset of labor was spontaneous in 74.7% deliveries, induced in 18.9%, and 4.3% had elective cesarean sections. Information for onset on labor was missing in 2.1% due to delivery in other hospitals. Labor resulted in spontaneous vaginal birth in 78.5%, vacuum extraction in 6.9%, emergency cesarean section in 10.2%, and information was missing for 4.4% due to delivery in other hospitals. The total incidence of HDP was 16.6% (96/577), of which 73 (12.6%) had gestational hypertension and 23 (4.0%) had PE. In total 12 (2.1%) women developed HDP before 37 weeks and 84 (14.5%) at ≥ 37 weeks with the vast majority of HDP 87.5% (84/96) occurring at term. Seven women delivered before 34 weeks of gestation, of which 3 because of PE (at gestational age 29 + 1, 29 + 4 and 32 + 3 weeks). One woman presented with gestational hypertension and was delivered prematurely due to acute placental abruption at 32 + 4 weeks. The causes of early preterm delivery in other remaining three cases included chorioamnionitis, intrauterine fetal death and cervical insufficiency, respectively. 20 women delivered between 34 and 37 gestational weeks, 5 of them had PE and 3 had gestational hypertension.

Other clinically significant pregnancy complications included acute appendicitis that required appendicectomy (*n* = 1), gestational diabetes (*n* = 3), intrahepatic cholestasis (*n* = 3), lung embolus (*n* = 1), premature pre-labour rupture of membranes (*n* = 4), pneumonia (*n* = 1), influenza (*n* = 1), and upper urinary tract infection (*n* = 1). 6.6% (38/577) of all newborns were SGA and had birth weight below 10 percentile based on Nordic birthweight reference standards^36^. In 6 of these, the mothers had gestational hypertension, none had PE, and all were delivered ≥ 37 + 0 gestational weeks. Total of 4.5% (26/577) of all newborns were reported to have had resuscitation at birth and 7.6% (44/577) of all newborns required admission to the neonatal intensive care unit for observation or treatment. One baby was diagnosed to have a dysmelic arm and another with infantile myofibromatosis after birth. There were two neonatal deaths; one baby delivered at 25 + 3 weeks of gestation died within 10 min of birth most likely due to acute chorioamnionitis, and another (born at 39 + 5 weeks) died due to disseminated intravascular coagulation. Other perinatal complications included prematurity (*n* = 1, gestational age at birth 33 + 1 weeks), hypoglycemia (*n* = 1), intracerebral hemorrhage and hydrocephalus (*n* = 1, gestational age at birth 32 + 4 weeks), pneumothorax (*n* = 1, born at gestational age 40 + 6), respiratory problems (*n* = 4, gestational age at birth 33 + 1, 29 + 4, 38 + 5 and 40 + 1), and meconium aspiration syndrome (*n* = 1, gestational age at birth 41 + 3). Remaining 32 neonates only required observation.

### Performance of the prediction model

**Fig. 1:**
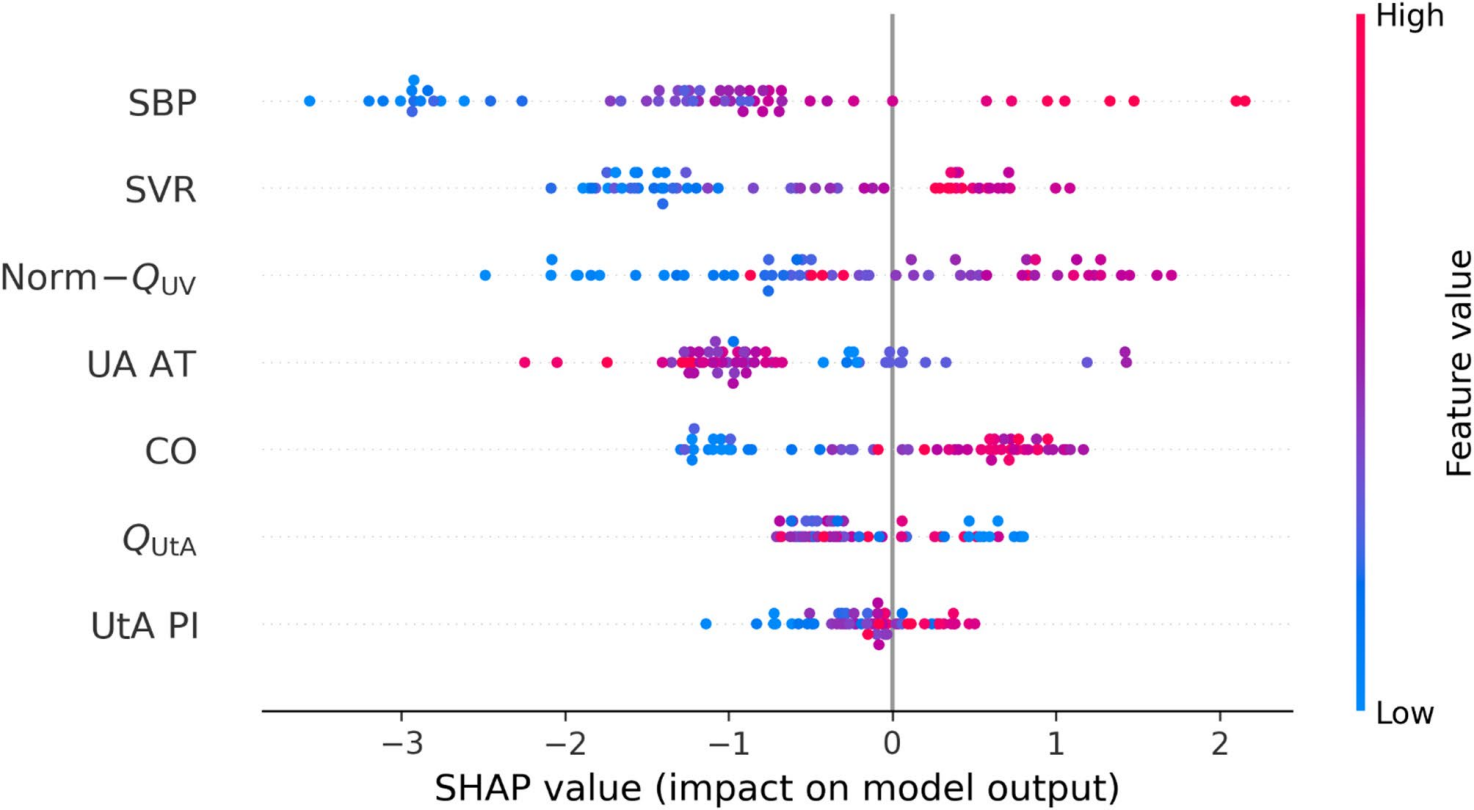
SHapley Additive exPlanations (SHAP) summary (beeswarm) on the independent test set. Global feature contributions to the predicted risk of hypertensive disorders of pregnancy (HDP) for the final XGBoost model using seven physiologically grounded predictors. Each point represents one participant; the horizontal position indicates the SHAP value (log-odds contribution to predicted HDP risk), and the color encodes the raw feature value (low → high). Features are ordered by their mean absolute SHAP value, indicating relative importance. Abbreviations: SBP, systolic blood pressure; SVR, systemic vascular resistance; CO, cardiac output; UA AT, umbilical artery acceleration time; UtA PI, mean uterine artery pulsatility index; Q_UtA_, total uterine artery blood flow (ml/min); norm-Q_UV,_ umbilical venous flow normalized to estimated fetal weight (ml/min/kg).

**Fig. 2:**
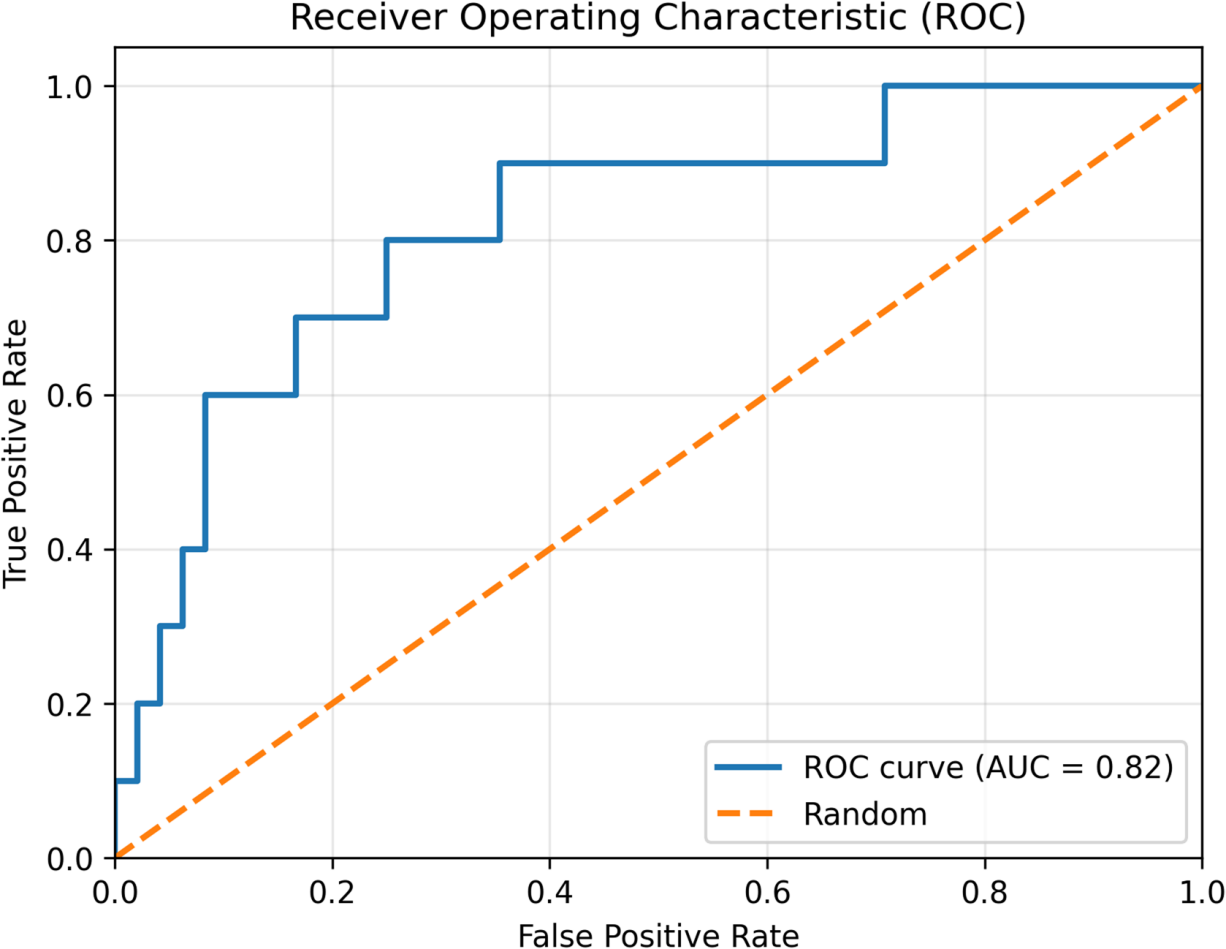
Receiver operating characteristic (ROC) curve for the independent test set. ROC curve of the final XGBoost model showing a ROC–AUC of 0.82 (95% CI, 0.65–0.95). The dashed diagonal line represents a non-informative (chance-level) classifier.

**Fig. 3:**
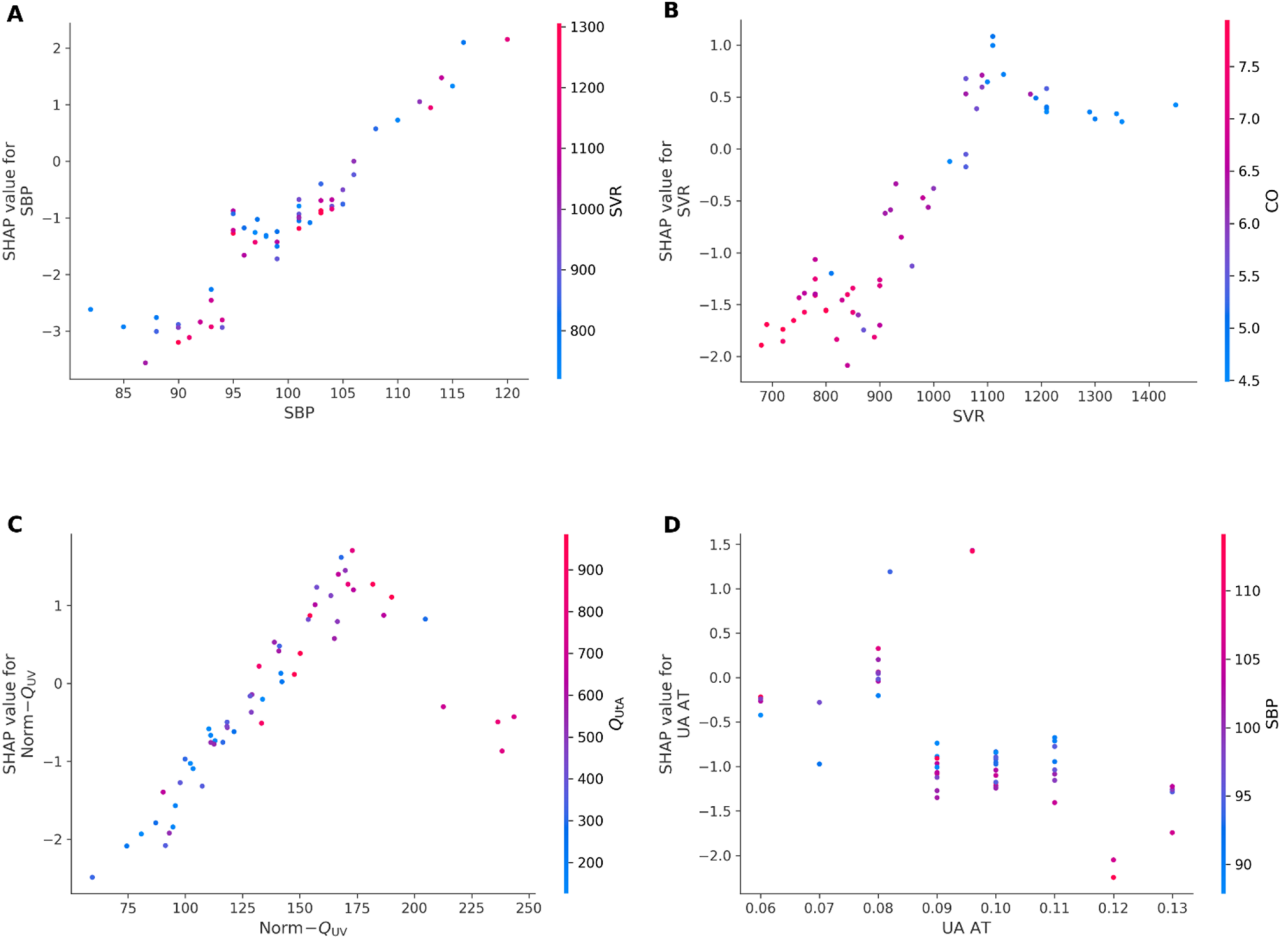
Shapley Additive Explanations (SHAP) dependence plots for the final XGBoost prediction model. Each point represents one participant from the independent test set. The *x*-axis shows the measured value of each feature, the *y*-axis shows its corresponding SHAP value (i.e., the feature’s contribution to the model’s predicted log-odds of hypertensive disorder of pregnancy [HDP]), and the color indicates the value of a physiologically related interacting variable. Positive SHAP values correspond to higher predicted HDP risk. **(A)** Systolic blood pressure (SBP): Higher SBP is strongly associated with increased predicted HDP risk, particularly at higher systemic vascular resistance (SVR) levels. **(B)** Systemic vascular resistance (SVR): Higher SVR corresponds to higher SHAP values, reflecting elevated risk; color-coding by cardiac output (CO) shows an inverse compensatory relationship. **(C)** Normalized umbilical venous flow (norm-Q_UV_) Reduced fetal venous flow is associated with increased HDP risk, while higher uterine blood flow (Q_UtA_) mitigates risk. **(D)** Umbilical artery acceleration time (UA AT): Shorter acceleration time is linked to higher predicted risk, particularly when combined with elevated SBP. These relationships illustrate physiologically plausible, often non-linear dependencies between maternal and fetoplacental hemodynamics and predicted HDP risk. Abbreviations: HDP, hypertensive disorders of pregnancy; SBP, systolic blood pressure; SVR, systemic vascular resistance; CO, cardiac output; UA AT, umbilical artery acceleration time; Q_UtA_ total uterine artery blood flow; norm-Q_UV_ normalized to estimated fetal weight (EFW).

The final predictive model developed using XGBoost incorporated seven physiologically grounded features with the highest SHAP importance values (Fig. 1). On the independent test set, model discrimination remained robust, achieving an ROC–AUC of 0.82 (95% CI 0.65–0.95) (Fig. 2). Among the four evaluated classifiers (details in Supplementary Table S2), XGBoost demonstrated the most balanced overall performance (G-mean = 0.74 [95% CI 0.56–0.89]), consistently outperforming CatBoost, LightGBM, and logistic regression in both discrimination and balanced accuracy. Using a validation-optimized operating point that maximized the G-mean of sensitivity and specificity, the model correctly identified 7 of 10 HDP cases (true positives = 7, false negatives = 3) and yielded 38 true negatives and 10 false positives. This operating point corresponded to a sensitivity of 70.1% (95% CI 39.9–100.0) and a specificity of 79.3% (95% CI 67.4–90.0). Although precision was moderate (40.9% [95% CI 17.6–64.7]), the model achieved a strong negative predictive value of 92.8% (95% CI 83.8–100.0) (Table 4). Overall performance metrics included accuracy of 77.8% (95% CI 67.2–87.9), F1-score 0.51 (95% CI 0.25–0.73), Matthews correlation coefficient 0.41 (95% CI 0.13–0.65), Brier score 0.12 (95% CI 0.06–0.18), and PR–AUC 0.57 (95% CI 0.26–0.83). All confidence intervals were derived from 1,000 bootstrap resamples to account for the limited number of positive cases (*n* = 10) in the test set. Finally, SHAP dependence plots for the top predictors (Fig. 3) illustrate how variations in individual features influence the predicted risk of HDP.

**Table 4:** Performance metrics for the final XGBoost model on the independent test set (*n* = 58).

Metric	Mean	95% Confidence Interval
Accuracy	77.8%	67.2%, 87.9%
Precision (PPV)	40.9%	17.6%, 64.7%
Recall (Sensitivity)	70.1%	39.9%, 100.0%
Specificity	79.3%	67.4%, 90.0%
F1-score	0.51	0.25, 0.73
MCC	0.41	0.13, 0.65
Brier score	0.12	0.06, 0.18
ROC–AUC	82.4%	64.8%, 95.1%
PR–AUC (AP)	56.8%	25.9%, 83.5%
G-mean	74.0%	55.5%, 89.4%

The operating threshold was determined on the validation set by maximizing the geometric mean (G-mean) of sensitivity and specificity and was subsequently applied unchanged to the independent test set. Values are presented as means with 95% bootstrap confidence intervals (1,000 resamples). At the chosen operating point, the confusion matrix counts were TP = 7, FN = 3, TN = 38, and FP = 10.

Abbreviations: G-mean, geometric mean; FN, false negatives; FP, false positives; MCC, Matthews correlation coefficient; PPV, positive predictive value; PR-AUC, Precision–Recall Area Under the Curve; ROC-AUC, Receiver Operating Characteristic – Area Under the Curve; TN, true negatives; TP, true positives.

Model interpretability was assessed using SHAP to quantify the relative contribution of each physiological feature to the model’s predictions (Fig. 3). The most influential predictors were SBP at mid-gestation (mean |SHAP| = 1.52), SVR (mean |SHAP| = 0.99), norm-Q_UV_; (mean |SHAP| =0.93), and UA AT (mean |SHAP| = 0.89). Maternal CO (mean |SHAP| = 0.74), Q_UtA_ (mean |SHAP| = 0.45), and UtA PI (mean |SHAP| = 0.29) contributed less strongly but remained consistent. Higher SBP and SVR were associated with increased predicted HDP risk, whereas higher CO and Q_UtA_ corresponded to lower predicted risk. These interpretable feature patterns align with established hemodynamic mechanisms in hypertensive pregnancy, underscoring both the clinical plausibility and transparency of the final XGBoost model.

## Discussion

Screening for HDP has been gradually adopted and implemented in clinical practice in several countries, however the detection rates remain modest, especially for the late-onset HDP, even when expensive biochemical tests are included in multivariate models^20^. A large study combining maternal characteristics, MAP, UtA PI, and placental growth factor (PlGF) at 11–13 weeks of gestation demonstrated a detection rate of 90%, 75%, and 41% for very early (requiring delivery at < 32 weeks), preterm (< 37 weeks), and term ($\:\ge\:$37 weeks) PE, respectively, at a 10% false positive rate^37^. The corresponding detection rates were reported to be 99%, 85% and 46%, respectively for screening at 19–24 weeks^13^.

Considering that cardiovascular maladaptation to pregnancy and placental dysfunction are the most important pathophysiological mechanisms in HDP, we aimed to develop a predictive model based solely on biophysical and hemodynamic markers of maternal cardiovascular function and placental circulation without any need for blood tests. 22–24 weeks of pregnancy was selected for the screening timepoint as the placentation and maternal physiological adaptation to pregnancy are mostly complete at this gestation. This timing also facilitates implementation of such a screening at the same visit as the second-trimester morphological ultrasound scan.

Our optimized predictive model incorporated seven physiologically grounded features identified through a data-driven selection process: maternal SBP, SVR, CO, Q_UtA_, UtA PI, norm-Q_UV_, and UA AT. Model performance was strong with mean accuracy of 77.8% and high specificity (79%) as well as sensitivity (70%). Although the precision (PPV) was moderate (41%), the negative predictive value (NPV) was high (92.8%), supporting the model’s suitability for screening applications where minimizing false negatives is critical. Mean F1-score reflected balanced classification and MCC indicated moderate-to-strong agreement between predictions and observed outcomes. A Brier score of 0.12 further indicated well-calibrated probability estimates. Compared to existing second-trimester screening strategies^13,38^, this approach demonstrated competitive performance particularly for term HDP without requiring any blood testing.

The final model was derived from an initial panel of 43 maternal and fetal hemodynamic features, spanning maternal cardiovascular hemodynamics, endothelial function, uterine, and umbilical blood flow domains. Predictors were retained based on their contribution to model discrimination (AUC and false negative rate) and their physiological relevance to maternal–placental adaptation. This iterative selection strategy produced a compact and interpretable model optimized for mid-pregnancy screening.

SHAP analysis confirmed that higher SBP at screening—even within normotensive ranges—remains a valuable predictor of HDP, aligning with findings from prior studies^39–41^. Other important predictive variables that featured in our model were maternal SVR and CO. These hemodynamic features capture maternal cardiovascular maladaptation to pregnancy, which is known to increase the risk of developing HDP. Notably, SVR emerged as the second most influential predictor, where higher resistance was consistently associated with increased risk underscoring the importance of increased maternal cardiac afterload in the pathophysiology of HDP.

A recent study in a Chinese population has shown that maternal CO is reduced, and SVR increased at 12–16 gestational weeks in women who develop PE later in pregnancy, but including these parameters did not improve the screening performance of currently used triple test (maternal MAP, UtA PI and placental growth factor)^42^. In contrast, our study showed that pregnant women who subsequently develop HDP had higher CO at 22–24 weeks compared to those who did not develop HDP. Although inconsistences in findings can be explained by differences in study population, study design, gestational age at screening, and outcome measures reported, our results are consistent with the findings of a large study of 4617 pregnant women evaluated by echocardiography in the first trimester^43^, as well as with that of another study on nulliparous women that reported similar observations^44^.

A shorter UA AT was associated with higher risk of developing HDP. To our knowledge, UA AT has not previously been identified as a predictive variable for HDP. It has been speculated that AT of arteries might be affected by CO, MAP, arterial compliance and blood viscosity, but no studies have confirmed this in the fetus. A small study demonstrated no association between UA AT measured within 2 days before birth and neonatal cord blood hematocrit^45^. Another cross-sectional study on 70 normal pregnancies at 18–40 weeks showed that UA AT is twice higher than the AT in the descending aorta at 18–23 weeks, decreasing with gestational age to be equalized in the late third trimester^46^. This might be due to gradual reduction in UA impedance and increase in placental blood flow with advancing gestation. Lower UA AT at 22–24 weeks may reflect a mechanism to compensate for reduced feto-placental blood flow that was identified as a risk factor for developing HDP in our model. This could be supported by the hypothesis of dynamic feto-placental blood flow matching with intervillous oxygen supply described by Talbert and Sebire^47^. However, further studies focusing on UA AT are needed to provide answers to unresolved questions.

It is interesting to note that the feto-placental (norm-Q_UV_) and utero-placental (Q_UtA_) volume blood flow parameters measured at 22–24 weeks were more powerful predictors of HDP compared to mean UtA PI, which is considered as a surrogate measure of uterine vascular resistance. These findings suggest that our model captures a nuanced interplay between maternal cardiovascular maladaptation and early signs of placental dysfunction and emphasize the importance of measuring volume blood flow to assess placental perfusion of both the maternal and the fetal sides of placenta.

Although endothelial dysfunction has been implicated in the pathomechanism of HDP, FMD, a gold standard noninvasive measure of endothelial function, did not feature as a factor in our prediction model. This may not be surprising as no previous study has confirmed its utility in screening for HDP^48^, although several studies have demonstrated decreased FMD in women with diagnosed PE ^48–52^.

From an implementation standpoint, all predictors on our best performing model are derived from easily obtainable biophysical measurements during the routine second trimester morphological scan visit. The utero-placental and feto-placental blood flow measurements can be performed at the same visit without increasing the scanning time significantly. Maternal BP can be easily measured and would not require an extra antenatal clinic visit. The CO can be measured with echocardiography instead of ICG, and sonographers could easily be trained to do it. Obtaining these measurements is not complicated and does not require extra equipment. This could obviate the need for serum/plasma biomarker testing which is expensive and not readily available in low resource settings.

A recent systematic review highlighted that imaging data as input data for machine learning techniques in predicting HDP is infrequently used^53^. The strength of our study is that it combines previously acknowledged maternal risk factors, such as BP, UtA Doppler, with the assessment of maternal systemic hemodynamics, and utero- and feto-placental blood flow parameters at gestational week 22 + 0–23 + 6 to develop an optimized prediction model for HDP using machine learning. Previous studies have found that there are significant differences between women developing HDP compared to those who do not, when assessed by some of these methods separately in the first or second trimester. However, we are not aware of any previous study that have used a combination of these measurements in predicting gestational hypertension or PE.

Our study participants were recruited from a population of pregnant women attending hospital for routine antenatal care including those with a personal or family history of PE unless they had preexisting hypertension or other medical conditions that are known to have high risk for developing HDP. In Norway, prevalence of HDP in a previous cohort has been reported to be 5.9%^4^ and in a patient-reported population-based study 15%^54^. In our cohort, HDP was detected in 16.6% of the participants, and 5 (0.87%) women required delivery before 34 weeks due to early onset HDP (three with PE and one gestational hypertension with abruptio placentae). The number of obese participants were lower than expected, as the pre-pregnancy obesity was reported to be 12.1% of all pregnant women in Norway in year 2016^55^. Therefore, selection bias was highly likely reflecting the single-center study design and patient recruitment from a tertiary care referral hospital with a different risk profile than the general population. We acknowledge that it may affect the generalizability of our findings and external validation is required to address this major limitation.

Another limitation is the lack of pre-pregnancy measurements to determine whether some women had predisposition to cardiovascular disease even though they did not have the diagnosis of chronic cardiovascular disease, as pre-pregnancy physiology has been shown to differ in women developing PE in future pregnancy^56^. A relatively low number of participants and the limited number of outcome events precluded subtype-specific analyses. Therefore, to test the validity of our prediction model for different types of HDP using stratification by gestational age or hemodynamic profiling was not possible. However, the vast majority of HDP occurred close to or at term, which highlights the reliability of model’s performance in predicting late onset disease.

Our results confirmed that maternal cardiovascular parameters were the most influential: higher SBP and higher SVR consistently increased the predicted risk of HDP. Notably, the model also revealed an inverse relationship for key feto-placental blood flow metrics, where lower Norm-Q_UV_ strongly contributed to higher predicted risk. The model’s discrimination on the independent test set was robust (ROC–AUC = 0.82), and at its G-mean–optimized operating point, it achieved high sensitivity (~ 70%) and an excellent negative predictive value (~ 93%), supporting its utility as a rule-out screening tool.

These results are competitive with existing second-trimester screening strategies that rely on maternal history, mean arterial pressure, UtA Doppler indices, and serum biomarkers. By integrating physiologically grounded interpretable features, this model offers a more comprehensive and accessible approach for identifying pregnancies at elevated risk for HDP—even in settings where biochemical testing is unavailable—potentially enabling earlier and more personalized clinical decision-making. Future studies should evaluate whether adding biochemical markers yields clinically meaningful incremental value.

## Supplementary Information

Below is the link to the electronic supplementary material.

Supplementary Material 1

## Data Availability

Anonymized data that support the findings of this study are available on request from the corresponding author, GA. The data are not publicly available due to regulatory restrictions to avoid circumstances that could compromise the privacy of research participants.

## Abbreviations

AUC: Area under the curve
AT: Acceleration time
BMI: Body mass index
CI: Confidence interval
CO: Cardiac output
FMD: Flow-mediated vasodilatation
HDP: Hypertensive disorders of pregnancy
MCC: Matthews correlation coefficient
PE: Pre-eclampsia
PI: Pulsatility index
SVR: Systemic vascular resistance
UA: Umbilical artery
UtA: Uterine artery
UV: Umbilical vein

## References

[1] Li, F. et al. Adverse pregnancy outcomes among mothers with hypertensive disorders in pregnancy: A meta-analysis of cohort studies. *Pregnancy Hypertens.* **24**, 107–117 (2021). 33813363 10.1016/j.preghy.2021.03.001

[2] Poon, L. C. et al. Hypertensive disorders of pregnancy and long-term cardiovascular health: FIGO best practice advice. *Int. J. Gynecol. Obstet.* **160**, 22–34 (2023). 10.1002/ijgo.14540 36635079

[3] Bastola, K., Koponen, P., Skogberg, N., Gissler, M. & Kinnunen, T. I. Hypertensive disorders of pregnancy among women of migrant origin in finland: A population-based study. *Acta Obstet. Gynecol. Scand.* **101**, 127–134 (2022). 34761373 10.1111/aogs.14291 PMC9564574

[4] Ebbing, C., Rasmussen, S., Skjærven, R. & Irgens, L. M. Risk factors for recurrence of hypertensive disorders of pregnancy, a population-based cohort study. *Acta Obstet. Gynecol. Scand.* **96**, 243–250 (2017). 27874979 10.1111/aogs.13066

[5] Sole, K. B., Staff, A. C., Räisänen, S. & Laine, K. Substantial decrease in preeclampsia prevalence and risk over two decades: A population-based study of 1,153,227 deliveries in Norway. *Pregnancy Hypertens.* **28**, 21–27 (2022). 35151209 10.1016/j.preghy.2022.02.001

[6] Wang, H. et al. Maternal hypertensive disorders and neurodevelopmental disorders in offspring: a population-based cohort in two nordic countries. *Eur. J. Epidemiol.* **36**, 519–530 (2021). 33948753 10.1007/s10654-021-00756-2 PMC8159819

[7] Eastabrook, G., Aksoy, T., Bedell, S., Penava, D. & de Vrijer, B. Preeclampsia biomarkers: an assessment of maternal cardiometabolic health. *Pregnancy Hypertens.* **13**, 204–213 (2018). 30177053 10.1016/j.preghy.2018.06.005

[8] Honigberg, M. C. et al. Polygenic prediction of preeclampsia and gestational hypertension. *Nat. Med.* **29**, 1540–1549 (2023). 37248299 10.1038/s41591-023-02374-9 PMC10330886

[9] Langen, I. et al. Hypertensive disorders of pregnancy among women with cardiovascular disease in norway: A historical cohort study. *Acta Obstet. Gynecol. Scand.* **103**, 1457 (2024). 38597240 10.1111/aogs.14841 PMC11168262

[10] Melchiorre, K., Giorgione, V. & Thilaganathan, B. The placenta and preeclampsia: villain or victim? *Am. J. Obstet. Gynecol.* **226**, S954–S962 (2022). 33771361 10.1016/j.ajog.2020.10.024

[11] Staff, A. C. The two-stage placental model of preeclampsia: an update. *J. Reprod. Immunol.* **134–135**, 1–10 (2019). 31301487 10.1016/j.jri.2019.07.004

[12] Bartsch, E., Medcalf, K. E., Park, A. L. & Ray, J. G. Clinical risk factors for pre-eclampsia determined in early pregnancy: systematic review and meta-analysis of large cohort studies. *BMJ* **353**, i1753 (2016). 27094586 10.1136/bmj.i1753 PMC4837230

[13] Gallo, D. M., Wright, D., Casanova, C., Campanero, M. & Nicolaides, K. H. Competing risks model in screening for preeclampsia by maternal factors and biomarkers at 19–24 weeks’ gestation. *Am. J. Obstet. Gynecol.* **214**, 619.e1-619.e17 (2016). 10.1016/j.ajog.2015.11.016 26627730

[14] North, R. A. et al. Clinical risk prediction for pre-eclampsia in nulliparous women: development of model in international prospective cohort. *BMJ* **342**, d1875 (2011). 21474517 10.1136/bmj.d1875 PMC3072235

[15] O’Gorman, N. et al. Multicenter screening for pre-eclampsia by maternal factors and biomarkers at 11–13 weeks’ gestation: comparison with NICE guidelines and ACOG recommendations. *Ultrasound Obstet. Gynecol.* **49**, 756–760 (2017). 28295782 10.1002/uog.17455

[16] Parra-Cordero, M. et al. Prediction of early and late pre-eclampsia from maternal characteristics, uterine artery doppler and markers of vasculogenesis during first trimester of pregnancy. *Ultrasound Obstet. Gynecol. Off J. Int. Soc. Ultrasound Obstet. Gynecol.* **41**, 538–544 (2013). 10.1002/uog.12264 22807133

[17] Wu, P., Green, M. & Myers, J. E. Hypertensive disorders of pregnancy. *BMJ* **381**, e071653 (2023). 37391211 10.1136/bmj-2022-071653

[18] Yang, Y. et al. Preeclampsia Prevalence, risk Factors, and pregnancy outcomes in Sweden and China. *JAMA Netw. Open.* **4**, e218401 (2021). 33970258 10.1001/jamanetworkopen.2021.8401 PMC8111481

[19] Coleman, M. A. G., McCowan, L. M. E. & North, R. A. Mid-trimester uterine artery doppler screening as a predictor of adverse pregnancy outcome in high-risk women. *Ultrasound Obstet. Gynecol.* **15**, 7–12 (2000). 10776006 10.1046/j.1469-0705.2000.00014.x

[20] Townsend, R. et al. Prediction of pre-eclampsia: review of reviews. *Ultrasound Obstet. Gynecol.* **54**, 16–27 (2019). 30267475 10.1002/uog.20117

[21] Rolnik, D. L. et al. Aspirin versus placebo in pregnancies at high risk for preterm preeclampsia. *N Engl. J. Med.* **377**, 613–622 (2017). 28657417 10.1056/NEJMoa1704559

[22] von Dadelszen, P., Syngelaki, A., Akolekar, R., Magee, L. A. & Nicolaides, K. H. Preterm and term pre-eclampsia: relative burdens of maternal and perinatal complications. *BJOG Int. J. Obstet. Gynaecol.* **130**, 524–530 (2023). 10.1111/1471-0528.17370 36562190

[23] Clark, A. R., James, J. L., Stevenson, G. N. & Collins, S. L. Understanding abnormal uterine artery doppler waveforms: A novel computational model to explore potential causes within the utero-placental vasculature. *Placenta* **66**, 74–81 (2018). 29884305 10.1016/j.placenta.2018.05.001 PMC6511649

[24] Pijnenborg, R., Dixon, G., Robertson, W. B. & Brosens, I. Trophoblastic invasion of human decidua from 8 to 18 weeks of pregnancy. *Placenta* **1**, 3–19 (1980). 7443635 10.1016/s0143-4004(80)80012-9

[25] Duvekot, J. J. & Peeters, L. L. Maternal cardiovascular hemodynamic adaptation to pregnancy. *Obstet. Gynecol. Surv.* **49**, S1–14 (1994). 7877788 10.1097/00006254-199412011-00001

[26] Du Bois, D. & Du Bois, E. F. A formula to estimate the approximate surface area if height and weight be known. *Nutr. Burbank Los Angel. Cty. Calif* **5**, 303–311; discussion 312–313 (1989). 2520314

[27] Vårtun, Å., Flo, K., Wilsgaard, T. & Acharya, G. Maternal functional hemodynamics in the second half of pregnancy: a longitudinal study. *PloS One*. **10**, e0135300 (2015). 26258418 10.1371/journal.pone.0135300 PMC4530890

[28] Corretti, M. C. et al. Guidelines for the ultrasound assessment of endothelial-dependent flow-mediated vasodilation of the brachial artery: a report of the international brachial artery reactivity task force. *J. Am. Coll. Cardiol.* **39**, 257–265 (2002). 11788217 10.1016/s0735-1097(01)01746-6

[29] Flo, K. et al. A longitudinal study of maternal endothelial function, inflammatory response and uterine artery blood flow during the second half of pregnancy. *Acta Obstet. Gynecol. Scand.* **95**, 225–232 (2016). 26462064 10.1111/aogs.12802

[30] Flo, K., Wilsgaard, T. & Acharya, G. Relation between utero-placental and feto-placental circulations: a longitudinal study. *Acta Obstet. Gynecol. Scand.* **89**, 1270–1275 (2010). 20726828 10.3109/00016349.2010.512069

[31] Acharya, G., Wilsgaard, T., Berntsen, G. K. R., Maltau, J. M. & Kiserud, T. Reference ranges for serial measurements of umbilical artery doppler indices in the second half of pregnancy. *Am. J. Obstet. Gynecol.* **192**, 937–944 (2005). 15746695 10.1016/j.ajog.2004.09.019

[32] Acharya, G., Wilsgaard, T., Rosvold Berntsen, G. K., Maltau, J. M. & Kiserud, T. Reference ranges for umbilical vein blood flow in the second half of pregnancy based on longitudinal data. *Prenat Diagn.* **25**, 99–111 (2005). 15712315 10.1002/pd.1091

[33] Flo, K., Wilsgaard, T. & Acharya, G. Agreement between umbilical vein volume blood flow measurements obtained at the intra-abdominal portion and free loop of the umbilical cord. *Ultrasound Obstet. Gynecol.* **34**, 171–176 (2009). 19606469 10.1002/uog.6441

[34] Hadlock, F. P., Harrist, R. B., Sharman, R. S., Deter, R. L. & Park, S. K. Estimation of fetal weight with the use of head, body, and femur measurements–a prospective study. *Am. J. Obstet. Gynecol.* **151**, 333–337 (1985). 3881966 10.1016/0002-9378(85)90298-4

[35] Barnett, S. B., Maulik, D. & International Perinatal Doppler Society. Guidelines and recommendations for safe use of doppler ultrasound in perinatal applications. *J. Matern Fetal Med.* **10**, 75–84 (2001). 11392597 10.1080/714904312

[36] Skjærven, R., Gjessing, H. K. & Bakketeig, L. S. Birthweight by gestational age in Norway. *Acta Obstet. Gynecol. Scand.* **79**, 440–449 (2000). 10857867

[37] Tan, M. Y. et al. Comparison of diagnostic accuracy of early screening for pre-eclampsia by NICE guidelines and a method combining maternal factors and biomarkers: results of SPREE. *Ultrasound Obstet. Gynecol. Off J. Int. Soc. Ultrasound Obstet. Gynecol.* **51**, 743–750 (2018). 10.1002/uog.19039 29536574

[38] Al-Amin, A. et al. Accuracy of second trimester prediction of preterm preeclampsia by three different screening algorithms. *Aust N Z. J. Obstet. Gynaecol.* **58**, 192–196 (2018). 28850663 10.1111/ajo.12689

[39] Hermida, R. C. et al. Blood pressure patterns in normal Pregnancy, gestational Hypertension, and preeclampsia. *Hypertension* **36**, 149–158 (2000). 10948070 10.1161/01.hyp.36.2.149

[40] Macdonald-Wallis, C. et al. Blood pressure change in normotensive, gestational hypertensive, preeclamptic and essential hypertensive pregnancies. *Hypertension* **59**, 1241 (2012). 22526257 10.1161/HYPERTENSIONAHA.111.187039 PMC3378662

[41] Magee, L. A. et al. The 2021 international society for the study of hypertension in pregnancy classification, diagnosis & management recommendations for international practice. *Pregnancy Hypertens.* **27**, 148–169 (2022). 35066406 10.1016/j.preghy.2021.09.008

[42] Wang, X. et al. Prediction of pre-eclampsia using maternal hemodynamic parameters at 12 + 0 to 15 + 6 weeks. *Ultrasound Obstet. Gynecol.* **65**, 173–182 (2025). 39825806 10.1002/uog.29177 PMC11788463

[43] De Paco, C., Kametas, N., Rencoret, G., Strobl, I. & Nicolaides, K. H. Maternal cardiac output between 11 and 13 weeks of gestation in the prediction of preeclampsia and small for gestational age. *Obstet. Gynecol.* **111**, 292 (2008). 18238965 10.1097/01.AOG.0000298622.22494.0c

[44] Khaw, A., Kametas, N. A., Turan, O. M., Bamfo, J. E. & Nicolaides, K. H. Maternal cardiac function and uterine artery doppler at 11–14 weeks in the prediction of pre-eclampsia in nulliparous women. *BJOG Int. J. Obstet. Gynaecol.* **115**, 369–376 (2008). 10.1111/j.1471-0528.2007.01577.x 18190374

[45] Chang, F. M. et al. Acceleration time in normal fetal umbilical artery at term and its relationship to the cord blood hematocrit. *J. Clin. Ultrasound JCU*. **23**, 33–37 (1995). 7699091 10.1002/jcu.1870230107

[46] Nishimoto, S. et al. Acceleration time of fetal arterial blood flow velocity waveforms: a preliminary study. *Osaka City Med. J.* **55**, 29–34 (2009). 19725432

[47] Talbert, D. & Sebire, N. J. The dynamic placenta: I. Hypothetical model of a placental mechanism matching local fetal blood flow to local intervillus oxygen delivery. *Med. Hypotheses*. **62**, 511–519 (2004). 15050098 10.1016/j.mehy.2003.10.025

[48] van Lopes, V. A. et al. Physiological adaptation of endothelial function to pregnancy: systematic review and meta-analysis. *Ultrasound Obstet. Gynecol.* **50**, 697–708 (2017). 28170124 10.1002/uog.17431

[49] Brandão, A. H. F., Félix, L. R., Patrício, E. C., Leite, H. V. & Cabral, A. C. V. Difference of endothelial function during pregnancies as a method to predict preeclampsia. *Arch. Gynecol. Obstet.* **290**, 471–477 (2014). 24748339 10.1007/s00404-014-3243-3

[50] Kamat, R., Jain, V. & Bahl, A. Serial Estimation of flow mediated dilatation in women at risk of hypertensive disorders of pregnancy. *Int. J. Cardiol.* **149**, 17–22 (2011). 20061037 10.1016/j.ijcard.2009.11.034

[51] Malhotra, A. S. et al. Serial profile of flow-mediated dilatation in primigravida for prediction of preeclampsia and gestational hypertension. *Hypertens. Pregnancy*. **37**, 212–219 (2018). 30273074 10.1080/10641955.2018.1524480

[52] Mannaerts, D. et al. Low-flow mediated constriction as a marker of endothelial function in healthy pregnancy and preeclampsia: A pilot study. *Pregnancy Hypertens.* **17**, 75–81 (2019). 31487661 10.1016/j.preghy.2019.02.001

[53] Zapata, R. D. et al. AI in hypertensive disorders of pregnancy: review. *Am. J. Hypertens.* **hpaf052**10.1093/ajh/hpaf052 (2025). 10.1093/ajh/hpaf052 40202855

[54] Andersgaard, A. B. et al. Recurrence and long-term maternal health risks of hypertensive disorders of pregnancy: a population-based study. *Am. J. Obstet. Gynecol.* **206**, 143e1–143e8 (2012). 10.1016/j.ajog.2011.09.032 22036665

[55] Nilsen, R. M. et al. Pre-pregnancy obesity among immigrant and non-immigrant women in norway: Prevalence, trends, and subgroup variations. *Acta Obstet. Gynecol. Scand.* **103**, 2081–2091 (2024). 39046200 10.1111/aogs.14923 PMC11426212

[56] Bernstein, I. M., Badger, G. J. & McBride, C. A. Prepregnancy physiology and subsequent preterm preeclampsia. *Am. J. Obstet. Gynecol.* **232**, 314.e1-314.e12 (2025). 10.1016/j.ajog.2024.05.031 PMC11584342 38789071

