# Supplementary material for: Development and validation of a novel prediction model for hypertensive disorders of pregnancy based on maternal cardiovascular function and placental blood flow metrics at 22 to 24 gestational weeks using machine learning
Source: Sci Rep. 2025 Dec 9;15:43488. doi: 10.1038/s41598-025-30738-3 (PMC12695884; doi:10.1038/s41598-025-30738-3)
Supplement: Supplementary file 1 — Supplementary Material 1 [file 41598_2025_30738_MOESM1_ESM.docx]

**Development and validation of a novel prediction model for hypertensive disorders of pregnancy based on maternal cardiovascular function and placental blood flow metrics at 22 to 24 gestational weeks using machine learning**

**Lantto Juulia**^1^**, Flo Kari^2,3^, Vårtun Åse^2^, Widnes Christian^2,4^, Johnson Jonas**^1^**, Acharya Ganesh**^1,2*^

**Supplementary Table S1. Original data on maternal endothelial function, cardiac function and systemic hemodynamics, utero-placental and feto-placental hemodynamics measured at 22+0 – 23+6 weeks of gestation.**

Data are presented as mean, standard deviation, 95% confidence intervals and difference between groups (women developing HDP vs. not developing HDP). Values presented for all women, women developing HDP and not developing HDP, respectively.

**Abbreviations:** ACI, accelerated cardiac index; BMI, body mass index; BP, blood pressure; CI, cardiac index; CO, cardiac output; HDP, hypertensive disorders of pregnancy; LCWI, left cardiac work index; LVET, left-ventricular ejection time; PEP, pre-ejection period; Q_UtA_, total uterine artery blood flow; norm-Q_UV_, volume blood flow in umbilical vein normalized for estimated fetal weight; R_UtA_ , uterine artery vascular resistance; STR, systolic time ratio; SVR, systemic vascular resistance; TFC, thoracic fluid content; UA PI, umbilical artery pulsatility index; UA RI, umbilical artery resistive index; UAAT, umbilical artery acceleration time; UA_Max_, umbilical artery maximum velocity; UA_Min_, end-diastolic flow in umbilical artery; UA_TAMX_, umbilical artery time-averaged maximum velocity; UtA PI, uterine artery pulsatility index; UtA RI, uterine artery resistive index; UtAAT, uterine artery acceleration time; UtA_Diam_, uterine artery diameter; UtA_Max_, uterine artery maximum velocity; UtA_Min_, uterine artery minimum velocity; UtA_TAMX_, uterine artery time-averaged maximum velocity; UtA_TAV_, uterine artery time-averaged velocity; UV_Diam_, umbilical vein diameter; VI, velocity index; ΔBADIA, change in brachial artery diameter in FMD.

|  | All women, N = 577 | | | | Women with HDP, N = 96 | | | | Women without HDP, N = 461 | | | | Difference between groups |
| --- | --- | --- | --- | --- | --- | --- | --- | --- | --- | --- | --- | --- | --- |
| Parameter | mean | SD | 95% CI, lower limit | 95% CI, upper limit | mean | SD | 95% CI, lower limit | 95% CI, upper limit | mean | SD | 95% CI, lower limit | 95% CI, upper limit | p-value |
| Maternal baseline characteristics at 22+0 – 23+6 weeks | | | | | | | | | | | | |  |
| Systolic BP (mmHg) | 101 | 9 | 101 | 102 | 108 | 9 | 106 | 110 | 100 | 8 | 99 | 101 | <0.001 |
| Diastolic BP (mmHg) | 68 | 7 | 67 | 68 | 72 | 7 | 71 | 74 | 70 | 7 | 66 | 67 | <0.001 |
| Mean arterial pressure (mmHg) | 79 | 7 | 78 | 80 | 84 | 7 | 83 | 86 | 78 | 7 | 77 | 79 | <0.001 |
| BMI (Kg/m^2^) | 23.94 | 3.34 | 23.66 | 24.21 | 24.81 | 3.67 | 24.06 | 25.55 | 23.76 | 3.25 | 23.47 | 24.05 | 0.007 |
| Maternal endothelial function at 22+0 – 23+6 weeks | | | | | | | | | | | | |  |
| ΔBADIA (mm) | 0.04 | 0.03 | 0.03 | 0.04 | 0.04 | 0.02 | 0.03 | 0.04 | 0.04 | 0.03 | 0.03 | 0.04 | 0.583 |
| Utero-placental blood flow measurements at 22+0 – 23+6 weeks | | | | | | | | | | | | |  |
| UtA_Diam_ (cm) | 0.37 | 0.07 | 0.36 | 0.38 | 0.37 | 0.07 | 0.36 | 0.39 | 0.37 | 0.07 | 0.36 | 0.38 | 0.784 |
| UtA_TAV_ (cm/s) | 36.12 | 12.60 | 35.09 | 37.15 | 37.27 | 13.29 | 34.58 | 39.96 | 35.90 | 12.46 | 34.78 | 37.01 | 0.314 |
| UtA_Max_ (cm/s) | 102.8 | 30.9 | 100.3 | 105.3 | 103.8 | 30.3 | 97.7 | 110.0 | 102.6 | 31.0 | 99.8 | 105.4 | 0.716 |
| UtA_Min_ (cm/s) | 49.3 | 16.3 | 48.0 | 50.7 | 48.9 | 18.0 | 45.2 | 52.5 | 49.4 | 15.9 | 48.0 | 50.9 | 0.810 |
| UtA_TAMX_ (cm/s) | 65.7 | 20.4 | 64.0 | 67.3 | 65.8 | 21.7 | 61.4 | 70.2 | 65.7 | 20.1 | 63.9 | 67.5 | 0.716 |
| UtA PI | 0.78 | 0.19 | 0.76 | 0.80 | 0.81 | 0.21 | 0.77 | 0.86 | 0.77 | 0.19 | 0.76 | 0.79 | 0.088 |
| UtA RI | 0.51 | 0.08 | 0.50 | 0.51 | 0.52 | 0.09 | 0.50 | 0.54 | 0.50 | 0.08 | 0.50 | 0.51 | 0.108 |
| UtAAT (s) | 0.11 | 0.02 | 0.11 | 0.11 | 0.11 | 0.02 | 0.11 | 0.11 | 0.11 | 0.02 | 0.11 | 0.12 | 0.107 |
| Q_UtA_ (ml/min) | 522.5 | 287.0 | 499.1 | 546.0 | 560.3 | 303.4 | 498.8 | 621.7 | 515.0 | 283.4 | 489.6 | 540.4 | 0.299 |
| R_UtA (mmHg/mL/min)_ | 0.20 | 0.11 | 0.19 | 0.21 | 0.20 | 0.13 | 0.18 | 0.23 | 0.20 | 0.11 | 0.19 | 0.21 | 0.810 |
| CO/Q_Ut_ (%) | 8.73 | 4.65 | 8.35 | 9.11 | 8.75 | 4.69 | 7.80 | 9.70 | 8.73 | 4.65 | 8.31 | 9.15 | 0.770 |
| Feto-placental blood flow measurements at 22+0 – 23+6 weeks | | | | | | | | | | | | |  |
| UA_Max_ (cm/s) | 35.47 | 5.20 | 35.05 | 35.90 | 34.62 | 5.40 | 33.53 | 35.72 | 35.64 | 5.15 | 35.18 | 36.10 | 0.069 |
| UA_MIN_ (cm/s) | 10.11 | 2.28 | 9.93 | 10.30 | 10.19 | 2.30 | 9.72 | 10.65 | 10.10 | 2.28 | 9.90 | 10.30 | 0.752 |
| UA_TAMX_ (cm/s) | 21.80 | 3.64 | 21.51 | 22.10 | 21.39 | 3.59 | 20.66 | 22.12 | 21.89 | 3.65 | 21.56 | 22.21 | 0.213 |
| UA PI | 1.17 | 0.14 | 1.16 | 1.18 | 1.16 | 0.14 | 1.13 | 1.19 | 1.18 | 0.14 | 1.16 | 1.19 | 0.205 |
| UA RI | 0.72 | 0.05 | 0.71 | 0.72 | 0.71 | 0.05 | 0.71 | 0.72 | 0.72 | 0.05 | 0.72 | 0.72 | 0.349 |
| UAAT (s) | 0.09 | 0.02 | 0.09 | 0.10 | 0.09 | 0.02 | 0.09 | 0.09 | 0.10 | 0.02 | 0.09 | 0.10 | 0.001 |
| UV_Diam_ (cm) | 0.46 | 0.05 | 0.45 | 0.46 | 0.47 | 0.05 | 0.46 | 0.48 | 0.45 | 0.05 | 0.45 | 0.46 | 0.032 |
| norm-Q_UV_ (ml/min per kg EFW) | 138.2 | 42.3 | 134.7 | 141.6 | 148.3 | 39.1 | 140.4 | 156.2 | 136.2 | 42.6 | 132.4 | 140.0 | 0.004 |
| Maternal cardiac function and systemic hemodynamics at 22+0 – 23+6 weeks | | | | | | | | | | | | | |
| CO (L/min) | 6.02 | 1.20 | 5.92 | 6.12 | 6.42 | 1.19 | 6.18 | 6.66 | 5.94 | 1.19 | 5.83 | 6.05 | <0.001 |
| CI (L/min/m^2^) | 3.33 | 0.51 | 3.28 | 3.37 | 3.48 | 0.51 | 3.38 | 3.59 | 3.30 | 0.50 | 3.25 | 3.34 | 0.003 |
| SVR  (dyne s/cm^5^) | 1027.4 | 194.6 | 1011.4 | 1043.3 | 1023.13 | 193.67 | 983.88 | 1062.37 | 1028.19 | 194.98 | 1010.72 | 1045.66 | 0.570 |
| SV (mL) | 78.13 | 14.72 | 76.93 | 79.34 | 80.85 | 15.61 | 77.69 | 84.02 | 77.59 | 14.49 | 76.29 | 78.89 | 0.045 |
| TFC (1/kOhm) | 27.62 | 4.16 | 27.28 | 27.96 | 28.13 | 4.58 | 27.20 | 29.06 | 27.52 | 4.07 | 27.15 | 27.88 | 0.191 |
| ACI (1/100s^2^) | 126.51 | 40.75 | 123.17 | 129.84 | 124.94 | 44.13 | 116.00 | 133.88 | 126.82 | 40.08 | 123.23 | 130.41 | 0.682 |
| PEP (ms) | 83.11 | 14.79 | 81.90 | 84.32 | 82.47 | 15.97 | 79.24 | 85.71 | 83.24 | 14.56 | 81.93 | 84.54 | 0.699 |
| LVET (ms) | 263.5 | 30.9 | 261.0 | 266.0 | 257.5 | 31.6 | 251.1 | 263.9 | 264.7 | 30.6 | 262.0 | 267.4 | 0.040 |
| VI (1/1000 s) | 77.50 | 21.02 | 75.79 | 79.22 | 77.97 | 22.51 | 73.41 | 82.53 | 77.41 | 20.73 | 75.55 | 79.27 | 0.701 |
| STR (%) | 32.35 | 6.81 | 31.79 | 32.90 | 32.91 | 7.27 | 31.44 | 34.39 | 32.23 | 6.72 | 31.63 | 32.83 | 0.321 |

**Supplementary Table S2. Comparative performance of machine-learning models for prediction of hypertensive disorders of pregnancy (HDP).**

Performance was evaluated on the independent test set for XGBoost, CatBoost, LightGBM, and logistic regression under identical preprocessing, feature sets, and hyperparameter optimization procedures. Metrics are reported with 95% bootstrap confidence intervals (1,000 resamples). To ensure a fair and clinically oriented comparison, operating thresholds for all models were determined on the validation set by maximizing the geometric mean (G-mean) of sensitivity and specificity, and subsequently applied unchanged to the test set.

**Abbreviations:** G-mean, geometric mean; FN, false negatives; FP, false positives; MCC, Matthews correlation coefficient; PPV, positive predictive value; PR–AUC, Precision–Recall area under the curve; ROC–AUC, Receiver Operating Characteristic area under the curve; TN, true negatives; TP, true positives.

| **Metric** | **XGBoost** | **CatBoost** | **LightGBM** | **Logistic Regression** |
| --- | --- | --- | --- | --- |
| **Accuracy** | **0.778 [0.672–0.879]** | 0.724 [0.620–0.828] | 0.845 [0.741–0.931] | 0.607 [0.483–0.724] |
| **Precision (PPV)** | **0.409 [0.176–0.647]** | 0.345 [0.143–0.565] | 0.561 [0.143–1.000] | 0.218 [0.055–0.400] |
| **Recall (TPR)** | **0.701 [0.399–1.000]** | 0.701 [0.375–1.000] | 0.403 [0.100–0.750] | 0.510 [0.182–0.833] |
| **Specificity (TNR)** | **0.793 [0.674–0.900]** | 0.728 [0.596–0.848] | 0.936 [0.860–1.000] | 0.627 [0.489–0.759] |
| **F1-score** | **0.507 [0.250–0.727]** | 0.454 [0.214–0.667] | 0.454 [0.133–0.714] | 0.299 [0.080–0.514] |
| **MCC** | **0.406 [0.134–0.652]** | 0.337 [0.072–0.582] | 0.383 [0.022–0.687] | 0.104 [−0.169–0.369] |
| **Brier score** | **0.115 [0.057–0.180]** | 0.116 [0.055–0.186] | 0.139 [0.061–0.226] | 0.162 [0.113–0.218] |
| **ROC-AUC** | **0.824 [0.648–0.951]** | 0.819 [0.658–0.945] | 0.784 [0.597–0.933] | 0.723 [0.528–0.893] |
| **PR-AUC (AP)** | **0.568 [0.259–0.835]** | 0.568 [0.268–0.823] | 0.413 [0.182–0.689] | 0.322 [0.148–0.528] |
| **G-mean** | **0.740 [0.555–0.894]** | 0.710 [0.520–0.867] | 0.596 [0.303–0.831] | 0.555 [0.327–0.745] |
| **NPV** | **0.928 [0.838–1.000]** | 0.923 [0.833–1.000] | 0.884 [0.792–0.962] | 0.862 [0.737–0.971] |
| **FPR** | **0.207 [0.100–0.326]** | 0.272 [0.152–0.404] | 0.064 [0.000–0.140] | 0.373 [0.241–0.511] |
| TP | 7 | 7 | 4 | 5 |
| TN | 38 | 35 | 45 | 30 |
| FP | 10 | 13 | 3 | 18 |
| FN | 3 | 3 | 6 | 5 |

**Supplementary Table S3. Final hyperparameters of the XGBoost model for prediction of hypertensive disorders of pregnancy (HDP).**

The table lists the optimal parameter configuration selected through RandomizedSearchCV (200 configurations; stratified 10-fold cross-validation) optimizing the mean precision–recall area under the curve (PR–AUC) on training folds. The final model achieved a mean cross-validated receiver operating characteristic area under the curve (ROC–AUC) of 0.95.

**Abbreviations:** AUC, area under the curve; PR–AUC, precision–recall area under the curve; ROC–AUC, receiver operating characteristic area under the curve.

| **Parameter** | **Selected value** |
| --- | --- |
| max_depth | 5 |
| n_estimators | 2000 |
| learning_rate | 0.05 |
| min_child_weight | 1 |
| subsample | 0.85 |
| colsample_bytree | 0.70 |
| min_split_loss | 0.2 |
| reg_alpha | 0.1 |
| reg_lambda | 1.0 |
| scale_pos_weight | 0.98 |
